# The Ciliopathy Gene *Ftm/Rpgrip1l* Controls Mouse Forebrain Patterning via Region-Specific Modulation of Hedgehog/Gli Signaling

**DOI:** 10.1523/JNEUROSCI.2199-18.2019

**Published:** 2019-03-27

**Authors:** Abraham Andreu-Cervera, Isabelle Anselme, Alice Karam, Christine Laclef, Martin Catala, Sylvie Schneider-Maunoury

**Affiliations:** Sorbonne Université, Centre National de la Recherche Scientifique (CNRS) UMR7622, Institut national pour la Santé et la Recherche Médicale U1156, Institut de Biologie Paris Seine-Laboratoire de Biologie du Développement (IBPS-LBD), 75005 Paris, France

**Keywords:** ciliopathy, forebrain patterning, Hedgehog/Gli signaling, primary cilia, *Rpgrip1l*

## Abstract

Primary cilia are essential for CNS development. In the mouse, they play a critical role in patterning the spinal cord and telencephalon via the regulation of Hedgehog/Gli signaling. However, despite the frequent disruption of this signaling pathway in human forebrain malformations, the role of primary cilia in forebrain morphogenesis has been little investigated outside the telencephalon. Here we studied development of the diencephalon, hypothalamus and eyes in mutant mice in which the *Ftm/Rpgrip1l* ciliopathy gene is disrupted. At the end of gestation, *Ftm*^−/−^ fetuses displayed anophthalmia, a reduction of the ventral hypothalamus and a disorganization of diencephalic nuclei and axonal tracts. In *Ftm*^−/−^ embryos, we found that the ventral forebrain structures and the rostral thalamus were missing. Optic vesicles formed but lacked the optic cups. In *Ftm*^−/−^ embryos, *Sonic hedgehog* (*Shh*) expression was virtually lost in the ventral forebrain but maintained in the zona limitans intrathalamica (ZLI), the mid-diencephalic organizer. Gli activity was severely downregulated but not lost in the ventral forebrain and in regions adjacent to the *Shh*-expressing ZLI. Reintroduction of the repressor form of Gli3 into the *Ftm*^−/−^ background restored optic cup formation. Our data thus uncover a complex role of cilia in development of the diencephalon, hypothalamus and eyes via the region-specific control of the ratio of activator and repressor forms of the Gli transcription factors. They call for a closer examination of forebrain defects in severe ciliopathies and for a search for ciliopathy genes as modifiers in other human conditions with forebrain defects.

**SIGNIFICANCE STATEMENT** The Hedgehog (Hh) signaling pathway is essential for proper forebrain development as illustrated by a human condition called holoprosencephaly. The Hh pathway relies on primary cilia, cellular organelles that receive and transduce extracellular signals and whose dysfunctions lead to rare inherited diseases called ciliopathies. To date, the role of cilia in the forebrain has been poorly studied outside the telencephalon. In this paper we study the role of the *Ftm/Rpgrip1l* ciliopathy gene in mouse forebrain development. We uncover complex functions of primary cilia in forebrain morphogenesis through region-specific modulation of the Hh pathway. Our data call for further examination of forebrain defects in ciliopathies and for a search for ciliopathy genes as modifiers in human conditions affecting forebrain development.

## Introduction

The Hedgehog (Hh) pathway plays an essential role in forebrain patterning, as illustrated by its frequent perturbation in holoprosencephaly (HPE), a human condition defined as a defect in the formation of midline structures of the forebrain and face (Muenke and Beachy, 2001; Fernandes and Hébert, 2008). Null mouse mutants for *Sonic hedgehog* (*Shh*) display a HPE phenotype (Chiang et al., 1996) and studies involving gene inactivation in mouse, lineage tracing, and loss- and gain-of-function approaches in chick identified multiple, successive functions of the Hh pathway in the diencephalon, hypothalamus, and eyes (Furimsky and Wallace, 2006; Vue et al., 2009; Jeong et al., 2011; Alvarez-Bolado et al., 2012; Haddad-Tóvolli et al., 2012, 2015; Blaess et al., 2015; Zhang and Alvarez-Bolado, 2016).

In vertebrates, transduction of Hh/Gli signaling depends on primary cilia, microtubular organelles with sensory functions. In the developing CNS, primary cilia are essential for proper dorsoventral (DV) patterning of the spinal cord via modulating Hh signaling. Shh binds to its receptor Ptch1, which removes Ptch1 from the cilium and relieves the inhibition of the G-protein-coupled receptor Smoothened (Smo) by Ptch1. Hh signaling at the cilium leads to the translocation of the Gli transcription factors into the nucleus and their activation into Gli activator form (GliA). In the absence of ligand, Gli2 and Gli3 are targeted to the proteasome in a cilium-dependent manner, giving rise to short forms with transcriptional repressor activity, among which Gli3R is a particularly strong repressor. Thus, the primary cilium is essential for the production of both GliR and GliA forms (Goetz and Anderson, 2010). In the forebrain, functional primary cilia are required for correct DV patterning of the telencephalon (Willaredt et al., 2008; Stottmann et al., 2009; Besse et al., 2011; Benadiba et al., 2012; Willaredt et al., 2013; Laclef et al., 2015) and for the proliferation of granule cell precursors in the dentate gyrus (Han et al., 2008). Surprisingly, despite the essential function of Hh signaling in the forebrain, the role of primary cilia outside the telencephalon has been little explored (Willaredt et al., 2013).

In this paper we study the function of the *Ftm/Rpgrip1l* gene in the forebrain. *RPGRIP1L* is a causal gene in severe human ciliopathies with brain abnormalities, Meckel–Gruber syndrome (MKS5 OMIM 611561) and Joubert syndrome type B (JBTS7 OMIM 611560; Arts et al., 2007; Delous et al., 2007). The Rpgrip1l protein is enriched at the ciliary transition zone (TZ), a region at the base of the axoneme distal to the basal body involved in the control of ciliary protein entry and exit (Reiter et al., 2012). Rpgrip1l is essential for the TZ localization of many other ciliopathy proteins (Mahuzier et al., 2012; Reiter et al., 2012; Shi et al., 2017; Wiegering et al., 2018). Rpgrip1l is also required for proteasome activity at the cilium base and for autophagy (Gerhardt et al., 2015; Struchtrup et al., 2018).

*Ftm*^−/−^ mouse fetuses die at or shortly before birth with a ciliopathy phenotype (Delous et al., 2007; Vierkotten et al., 2007) and lack cilia in the developing telencephalon (Besse et al., 2011). Using this mutant, our laboratory has previously shown that primary cilia are required for telencephalic DV patterning. In *Ftm*^−/−^ embryos, the olfactory bulbs and corpus callosum, two dorsal telencephalic structures, are missing because of an expansion of the ventral telencephalon. The phenotype is rescued by introduction into the *Ftm* mutant of one allele of *Gli3*^Δ*699*^ (Besse et al., 2011; Laclef et al., 2015), which produces constitutively a short form of Gli3 with repressor activity (Hill et al., 2007). These studies demonstrate that the main role of cilia in telencephalic patterning is to permit Gli3R formation.

What is the role of primary cilia in other forebrain regions? Here we show that *Ftm*^−/−^ fetuses display severely disorganized hypothalamus and diencephalon and lack eyes. Investigating the molecular causes of these defects, we find that *Shh* expression and Hh signaling (hereafter called Shh signaling, Shh being the principal ligand in the forebrain) are differentially affected in different forebrain regions. Our results uncover essential and diverse functions for Ftm/Rpgrip1l and cilia in Gli activity in patterning the forebrain and eyes.

## Materials and Methods

### 

#### 

##### Mice.

All experimental procedures involving mice were made in agreement with the European Directive 2010/63/EU on the protection of animals used for scientific purposes, and the French application decree 2013-118. Mice were raised and maintained in the IBPS mouse facility, approved by the French Service for Animal Protection and Health, with the approval numbers C-75-05-24. The project itself has been approved by the local ethical committee “Comité d'éthique Charles Darwin”, under the authorization #2015052909185846. *Gli3*^**Δ***699*^- also named Gli3^Δ^- and *Ftm*-deficient mice were produced and genotyped as described previously (Böse et al., 2002; Besse et al., 2011). Mutant lines were maintained as heterozygous (*Ftm*^+/−^ or *Gli3*^**Δ***699*/+^) and double-heterozygous (*Ftm*^+/−^; *Gli3*^**Δ***699*/+^) animals in the C57BL/6J background. Note that the eye phenotype of the *Ftm*^−/−^ animals was totally penetrant in the C57BL/6J background used here, unlike in C3H or mixed backgrounds (Delous et al., 2007; Wiegering et al., 2018). The transgenic line Tg[GBS::GFP] was maintained in the C57BL/6J background and genotyped as described previously (Balaskas et al., 2012). In analyses of *Ftm* mutant phenotypes, heterozygous and wild-type (WT) embryos did not show qualitative differences, and both were used as “control” embryos. The sex of the embryos and fetuses was not analyzed. Embryonic day (E)0.5 was defined as noon on the day of vaginal plug detection.

##### Histology, ISH, and IF.

For whole-mount *in situ* hybridization (ISH), embryos were dissected in cold PBS and fixed in 4% paraformaldehyde (PFA) in PBS for a time depending on the embryonic age and then processed as described by Anselme et al. (2007). For histology and ISH on sections, embryos were dissected in cold PBS and fixed overnight in 60% ethanol, 30% formaldehyde and 10% acetic acid. Embryos were embedded in paraffin and sectioned (7 μm). Cresyl thionin staining and ISH were performed on serial sections, as described previously (Anselme et al., 2007; Besse et al., 2011; Laclef et al., 2015). For fluorescence ISH (FISH), immunodetection of the probe was done overnight at 4°C with anti-Digoxigenin peroxidase-conjugated antibody (Roche), diluted 1:50 in maleate buffer supplemented with 2% Boehringer Blocking Reagent (Roche). Peroxidase activity was detected with FITC-coupled tyramide (1:50).

For immunofluorescence (IF), embryos were fixed overnight in 4% PFA. E18.5 fetuses were perfused with 4% PFA. IF staining was performed on 14 μm serial cryostat sections, as described previously (Anselme et al., 2007; Laclef et al., 2015), with antibodies against Shh (Cell Signaling Technology, 2207; 1:200 and R&D Systems, AF445; 1:200), Arl13b (Neuromab 75-287; 1:1500), FoxA2 (Abcam, ab23630; 1:200), GFP (Aves, GFP-1020; 1:200), Rpgrip1l (Besse et al., 2011; 1:800), Mash1 (BD Pharmigen, 556604; 1:200), Neurofilament [Developmental Studies Hybridoma Bank (DSHB) 2H3; 1:200], Tuj1 (Sigma-Aldrich, T8578; 1:500), Tag1 (DSHB, 23.4-5; 1:50), and Robo3 (R&D Systems, AF3076; 1:200). Secondary antibodies were AlexaFluor conjugates from Invitrogen (1:1000). Nuclei were stained with DAPI (1:500).

##### DiI/DiA labeling.

**Figure 1.**
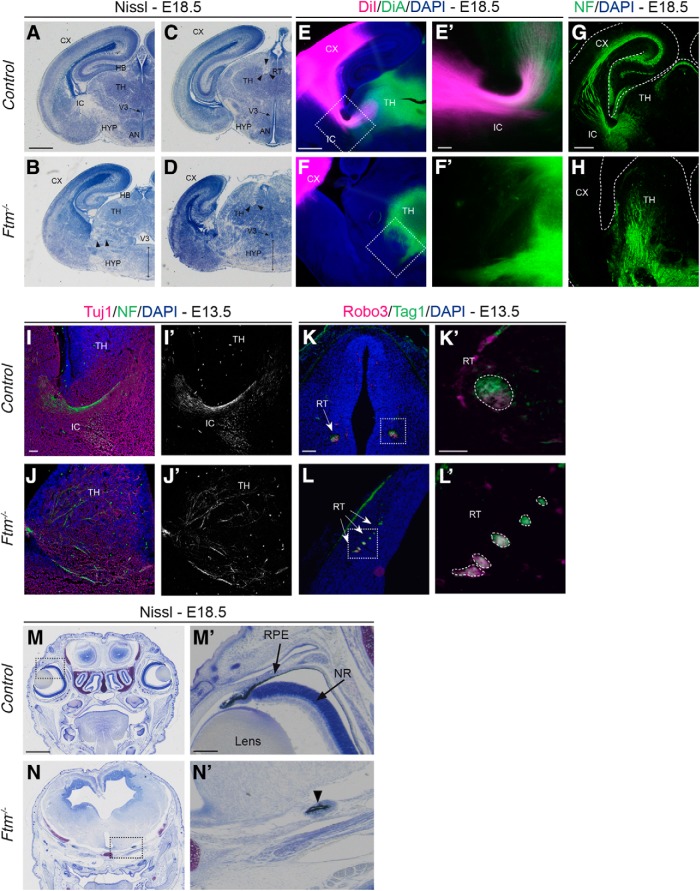
Histology and labeling of axon tracts in the brain of control and *Ftm*^−/−^ fetuses. ***A***–***D***, Nissl staining on coronal sections of the brain at two distinct anteroposterior levels of thalamic and hypothalamic regions in E18.5 WT (***A***, ***C***) and *Ftm*^−/−^ (***B***, ***D***) fetuses. ***C***, ***D***, More posterior sections than ***A*** and ***B***. Both levels of sections correspond to the ventral hypothalamus and the alar thalamus. Black arrowheads in ***B***–***D*** point to axon fascicles of the IC and RT. Double black arrows in ***B*** and ***D*** point to the dysmorphic hypothalamus in *Ftm* mutants. ***E***–***F′***, Carbocyanine dye staining of corticothalamic (DiI, magenta) and thalamocortical (DiA, green) axons in E18.5 WT (***E***, ***E′***) and *Ftm*^−/−^ (***F***, ***F′***) brains. ***E′***, ***F′***, Higher-magnification of the boxed regions in ***E*** and ***F***, respectively. ***G***, ***H***, Neurofilament (NF) immunostaining of axon tracts in E18.5 control (***G***) and *Ftm*^−/−^ (***H***) brains. ***I***–***L′***, Immunofluorescence for Tuj1 and NF (***I***–***J′***) and for Robo3 and Tag1 (***K***–***L′***) in E13.5 control (***K***, ***K′***) and *Ftm*^−/−^ (***L***, ***L′***) brains. ***M***–***N′***, Nissl staining on coronal sections at the level of the eyes of the head of E18.5 WT (***M***, ***M′***) and *Ftm*^−/−^ (***N***, ***N′***) fetuses. ***M′***, ***N′***, Higher-magnification of the boxed regions in ***M*** and ***N***, respectively. The arrowhead in ***N′*** points to remnants of the RPE. AN, Anteroventral nucleus; CX, cortex; HB, Habenula; NR, neural retina; V3 third ventricle. Scale bars: (in ***A***, ***M***) ***A***–***D***, ***M***, ***N***, 1 mm; (in ***E***, ***G***) ***E***–***H***, 0.5 mm; (in ***E′***, ***I***, ***K′***), ***E′***, ***F′***, ***I–J′***, ***K′***, ***L′***, 0.1 mm; (in ***K***, ***M′***) ***K***, ***L***, ***M′***, ***N′***, 0.2 mm.

Brains of E18.5 fetuses were dissected in PBS 1× and fixed overnight in 4% PFA. After three washes in PBS, brains were labeled by 1,1′-dioctadecyl-3,3,3′,3′-tetramethylindocarbocyanine perchlorate (DiI; Invitrogen D383) or 4-Di-16 ASP [4,4-(dihexadecylaminostyril)*-N-*methylpyridinium iodide (DiA); Invitrogen, D3883] crystals, in the cortex or in the diencephalon of control and *Ftm*^−/−^ brains, as indicated in Figure 1. Samples were kept for at least 2 weeks in PFA 4% at 37°C for the lipophilic dye to diffuse along the fixed cell membranes. Then, the brains were embedded in 4% agarose in PBS, and thick coronal vibratome (LeicaVT1000S) sections were made.

##### Image acquisition and quantification of fluorescence intensity.

ISH images were acquired with a bright-field Leica MZ16 stereomicroscope. IF, FISH, and axonal tract dye labeling images were observed with a fluorescent binocular (Leica, M165FC) and acquired with a confocal microscope (Leica, TCS SP5 AOBS).

Fluorescence intensity was measured using ImageJ software. For Shh-GFP immunofluorescence, adjacent squares of 50 μm side were drawn in the diencephalon, all along the ventricular surface from posterior to anterior. Total fluorescence intensity was measured in each square on three distinct optical sections. For each optical section, the background intensity was measured by taking three squares in the third ventricle, and the mean background intensity was subtracted from all the measurements of the same image. Images from three controls, three *Ftm*^−/−^, and two [*Ftm*^−/−^, *Gli3*^Δ/+^] embryos were used for quantification. For comparison, the measurements were aligned using as a reference the square corresponding to the AP level of the zona limitans intrathalamica (ZLI; point 6 of the ordinate in Fig. 7*P*, *Q*). The diagrams in Fig. 7*P*, *Q* indicate the mean intensity for each position of each genotype.

For quantification of *Ptch1* FISH, adjacent squares of 20 μm side were drawn in the diencephalon, from posterior to anterior, at two apico-basal levels: along the ventricular surface and ∼40 μm away from the ventricular surface. Total fluorescence intensity was measured in each square on three distinct optical sections. Images from four controls, four *Ftm*^−/−^, and three [*Ftm*^−/−^, *Gli3*^Δ/+^] embryos were used for quantification. For comparison, the measurements were aligned using as a reference the square corresponding to the AP level of the ZLI.

##### Scanning electron microscopy.

Embryos were dissected in 1.22× PBS, pH 7.4, 0.1 m sodium cacodylate, and fixed overnight with 2% glutaraldehyde in 0.61× PBS, pH 7.4, 0.1 m sodium cacodylate at 4°C. Heads were then sectioned to separate the left and right sides of the forebrain, exposing their ventricular surfaces. Head samples were washed several times in 1.22× PBS and postfixed for 15 min in 1.22× PBS containing 1% OsO_4_. Fixed samples were washed several times in ultrapure water, dehydrated with a graded series of ethanol, and critical point dried (CPD 300, Leica) at 79 bar and 38°C with liquid CO_2_ as the transition fluid and then depressurized slowly (0.025 bar/s). They were then mounted on aluminum mounts with conductive silver cement. Samples surfaces were coated with a 5 nm platinum layer using a sputtering device (ACE 600, Leica). Samples were observed under high vacuum conditions using a Field Emission Scanning Electron Microscope (Gemini 500, Zeiss) operating at 3 kV, with a 20 μm objective aperture diameter and a working distance ∼3 mm. Secondary electrons were collected with an in-lens detector. Scan speed and line compensation integrations were adjusted during observation.

##### Experimental design and statistical analysis.

In all experiments, the number of embryos or fetuses analyzed was ≥3 for each genotype, unless otherwise stated. For the comparison of the number of cilia in the different diencephalic regions of control and *Ftm*^−/−^ embryos in Figure 10*P*, quantification was made in four control and four mutant embryos. The number of sections (for cilia density) and of cilia (for cilium length) analyzed are indicated on the graph. In Figure 7, for the quantification of Shh and GFP fluorescence intensity (Fig. 7*P*,*Q*), we compared three control, three *Ftm*^−/−^, and two [*Ftm*^−/−^, *Gli3*^Δ/+^] embryos; for *Ptch1* intensity (Fig. 7*R*,*S*), we compared four control, four *Ftm*^−/−^, and three [*Ftm*^−/−^, *Gli3*^Δ/+^] embryos. Statistical analysis was performed using the Prism software. For cilia length (Fig. 10*P*, bottom graph), unpaired *t* test was performed. For cilia density (Fig. 10*P*, top graph) and for Figure 7*P–S*, we used a nonparametric tests (Figs. 10*P*, top, Mann–Whitney, 7*P–S*, Kruskal–Wallis) because the number of samples was too low to achieve normality of distribution. For Figure 7*P–S*, exact *p* values are available on request. For quantification of ZLI width, a Mann–Whitney test was used.

## Results

### *Ftm*^−/−^ fetuses at the end of gestation display microphthalmia and profound perturbations of the diencephalon and hypothalamus

Histological analysis combined with dye labeling and immunostaining of axonal tracts showed profound defects in the diencephalon and hypothalamus of *Ftm*^−/−^ fetuses at the end of gestation (E18.5; Fig. 1). The ventral regions of the diencephalon and hypothalamus were particularly affected, with a highly dysmorphic ventral part and a perturbed position and shape of the third ventricle (Fig. 1*A–D*). In wild-type fetuses, habenular and thalamic nuclei were clearly visible in the dorsal region (Fig. 1*A*,*C*). In *Ftm*^−/−^ fetuses, these nuclei were also present even if their organization was mildly perturbed (Fig. 1*B*,*D*). In contrast, the ventral brain appeared highly disorganized in *Ftm*^−/−^ fetuses (Fig. 1*A–D*). The ventral midline, normally thin in WT, was enlarged in *Ftm*^−/−^, likely because of the absence of the most ventral region and fusion of the lateral parts. The most medial hypothalamic nuclei (such as the anteroventral nuclei) were indistinguishable. The dorsal diencephalon and hypothalamus were present although malformed. In both regions, the axonal tracts [internal capsule (IC) and retroflexus tract (RT)] were disorganized in *Ftm*^−/−^ brains (Fig. 1*B*,*D*, arrowheads). Defects in corticothalamic (CTA) and thalamocortical (TCA) axonal tracts were confirmed with carbocyanine dye labeling (DiI and DiA, respectively) (Fig. 1*E–F′*). In WT brains, both CTA (magenta) and TCA (green) axons were visualized and colocalized in the IC (Fig. 1*E*,*E′*). In *Ftm*^−/−^ brains, neither CTA nor TCA grew sufficiently to reach the IC (Fig. 1*F*,*F′*). The disorganization of the TCA tracts was confirmed using neurofilament staining (Fig. 1*G*,*H*). In the E18.5 control brain (Fig. 1*G*), CTA axons met TCA axons in the IC. In the mutant, thalamic axons were mainly oriented ventrally and the IC was not detected (Fig. 1*H*). These axonal defects were confirmed at earlier developmental stages using Neurofilament staining and with Robo3 and Tag1 to label the retroflexus tract (Fig. 1*I–L′*). The eyes were absent in all *Ftm*^−/−^ fetuses (Fig. 1*M–N′*), only remnants of the retinal pigmented epithelium were observed under the brain (Fig. 1*N′*, arrowhead). We next focused on the developmental origin of these defects.

### Patterning of the diencephalon and hypothalamus is affected in *Ftm*^−/−^ embryos

**Figure 2.**
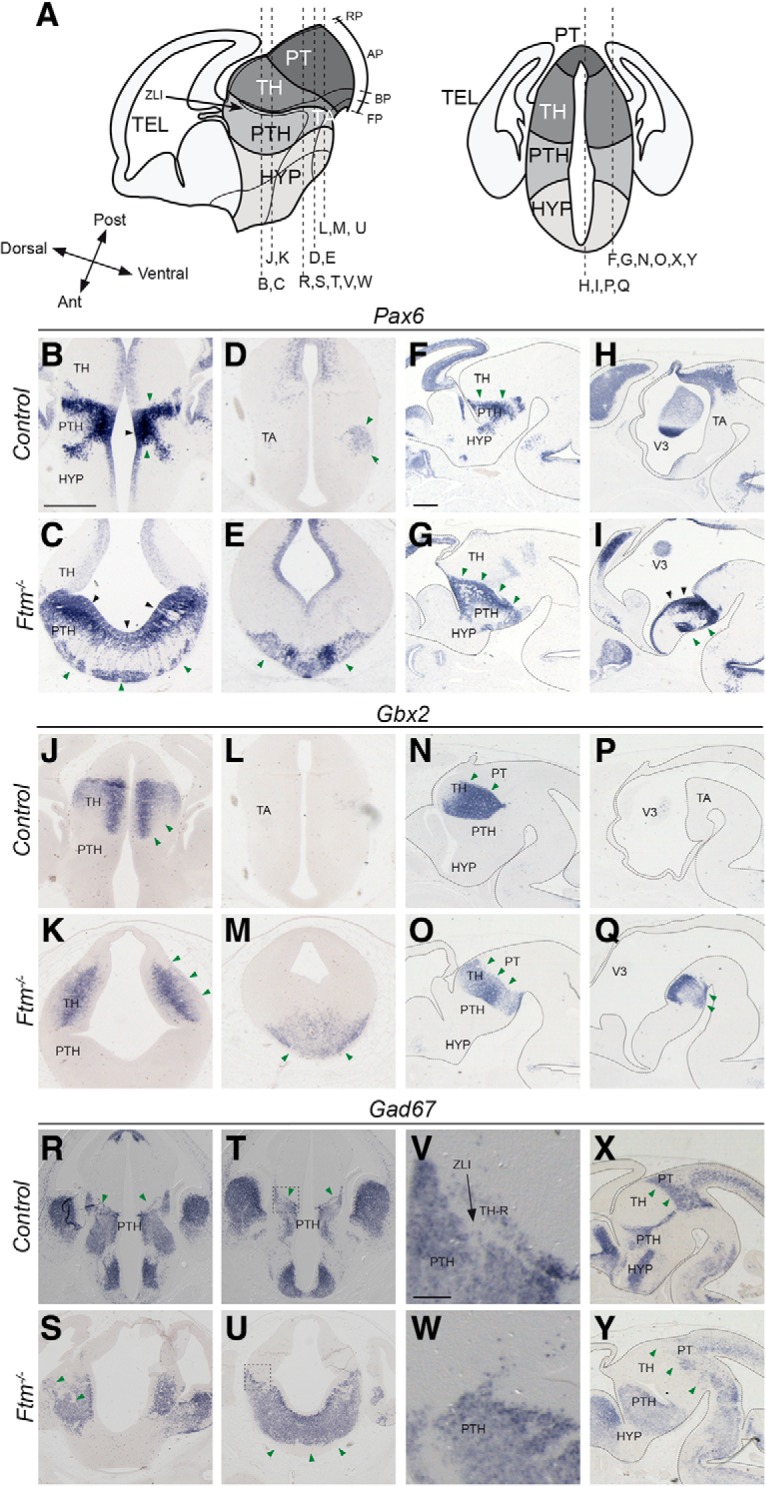
Diencephalon patterning at E13.5. ***A***, Schematic drawings of the E13.5 forebrain in sagittal (left) and coronal (right) views. The position of the coronal (***B***–***E***, ***J***–***M***, ***R***–***W***) and sagittal (***F***–***I***, ***N***–***Q***, ***X***, ***Y***) sections shown below is indicated with dashed lines. Note that in the left diagram, anteroposterior and dorsoventral axes are indicated at the level of the ZLI. ***B***–***Y***, ISH with probes for *Pax6* (***B***–***I***), *Gbx2* (***J***–***Q***), and *Gad67* (***R–Y***) in coronal sections at two distinct anteroposterior levels (***B***–***E***, ***J***–***M***, ***R***–***W***) and in sagittal sections at lateral (***F***, ***G***, ***N***, ***O***, ***X***, ***Y***) and medial (***H***, ***I***, ***P***, ***Q***) levels. The genotype (*control* or *Ftm*^−/−^) is indicated on the left, where control stands for *Ftm*^+/+^ or *Ftm*^+/−^. In sagittal sections, the brain is outlined with dotted lines. Black and green arrowheads point to neuronal progenitors and neurons, respectively. Ant, Anterior; BP, basal plate; FP, floor plate; RP, roof plate; Post, posterior; TEL, telencephalon; 3V, third ventricle. Scale bars: (in ***B*** for coronal sections, in ***F*** for sagittal sections), ***A***–***U***, ***X***, ***Y***, 0.5 mm; (in ***V***) ***V***, ***W***, 100 μm.

The developing diencephalon is subdivided along the DV axis in roof, alar, basal, and floor plates, and along the caudo-rostral axis in three regions or prosomeres, p1, p2, and p3. The alar plates of p1, p2, and p3 give rise to the pretectum (PT), thalamus (TH), and prethalamus (PTH), respectively (Fig. 2*A*). The ZLI is located at the junction between the TH and PTH. The ZLI acts as an organizer for the TH and PTH, regulating proliferation and cell fate in these two regions (Epstein, 2012; Hagemann and Scholpp, 2012; Zhang and Alvarez-Bolado, 2016).

**Figure 3.**
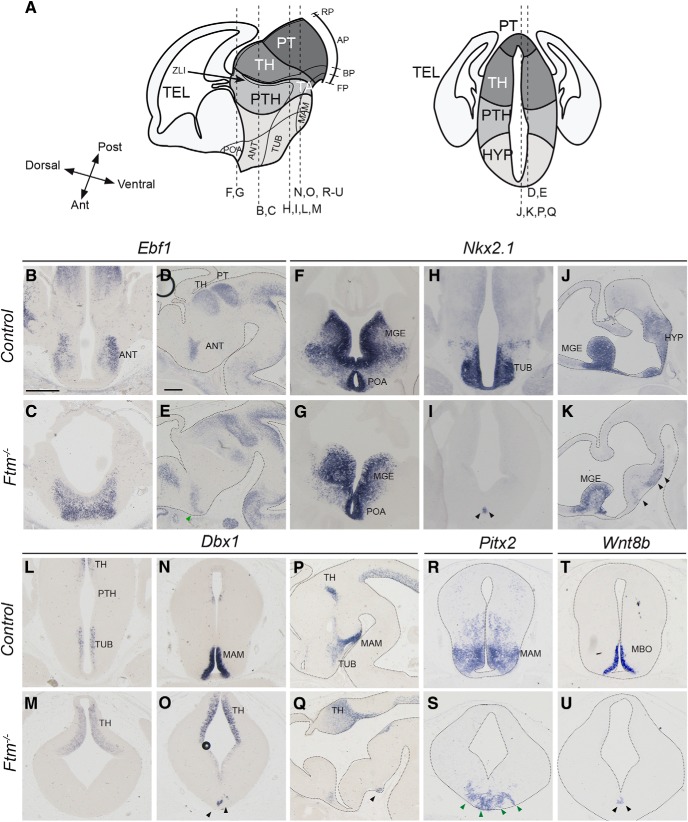
Hypothalamus patterning at E13.5. ***A***, Schematic drawings of the E13.5 forebrain in sagittal (left) and coronal (right) views. The position of the coronal (***B***, ***C***, ***F***–***I***, ***L***–***O***, ***R***–***U***) and sagittal (***D***, ***E***, ***J***, ***K***, ***P***, ***Q***) sections shown below is indicated with dashed lines. Note that in the left diagram, anteroposterior and dorsoventral axes are indicated at the level of the hypothalamus. ***B***–***U***, ISH with probes for *Ebf1* (***B–E***), *Nkx2.1* (***F–K***), *Dbx1* (***L–Q***), *Pitx2* (***R***, ***S***), and *Wnt8b* (***T***, ***U***) in coronal sections at different anteroposterior levels and in sagittal sections. The genotype (*control* or *Ftm*^−/−^) is indicated on the left. Black and green arrowheads point to neuronal progenitors and neurons, respectively. In sagittal sections and in coronal sections in ***R***–***U***, the brain is outlined with dotted lines. Ant, Anterior; AP, alar plate; BP, basal plate; FP, floor plate; MBO, mammillary body; Post, posterior; RP, roof plate; TEL, telencephalon. Scale bars: (in ***B*** for coronal sections, in ***D*** for sagittal sections), 0.5 mm.

To investigate diencephalon patterning in *Ftm* mutants, we performed ISH for genes expressed in these different regions, on coronal and sagittal sections of E13.5 embryos. We first used the alar plate-expressed genes *Pax6* (PTH), *Gbx2* (TH), and *Gad67* (PTH and PT; Fig. 2) encoding, respectively, two transcription factors involved at multiple steps of brain patterning and neurogenesis and a subunit of the glutamate decarboxylase involved in the synthesis of GABA (Stoykova and Gruss, 1994; Stoykova et al., 1996; Miyashita-Lin et al., 1999; Katarova et al., 2000; Hevner et al., 2002). We found that the expression domains of these genes were expanded along the DV axis in *Ftm*^−/−^ embryos (Fig. 2). In control embryos, robust *Pax6* expression was detected in both the ventricular (VZ) and subventricular zone of the PTH as well as in differentiating neuronal populations (Fig. 2*B*,*D*,*F*,*H*). *Pax6* was also more faintly expressed in the VZ of the adjacent regions. In *Ftm*^−/−^ embryos, we observed a ventral expansion of the *Pax6* expression domain, which now reached the ventral midline (Fig. 2*C*,*E*,*G*,*I*, green arrowheads). In addition, in anterior coronal sections, the hypothalamic, *Pax6*-negative region was absent from the sections shown (Fig. 2*C*). *Gbx2* expression in control embryos was observed in differentiating neurons of the TH but not in the tegmental areas (TAs) of the diencephalon (Fig. 2*J*,*L*,*N*,*P*). In *Ftm*^−/−^ embryos, *Gbx2* expression expanded ventrally (Fig. 2*K*,*M*,*O*,*Q*). *Gad67* expression in the control diencephalon was widespread in neurons of the PT and PTH and absent from diencephalic TA (Fig. 2*R*,*T*,*X*). In *Ftm*^−/−^ embryos, the PT and PTH expression domains expanded ventrally (Fig. 2*S*,*U*,*Y*). The ventral expansion of the diencephalic alar plate and the reduction of the basal plate in *Ftm*^−/−^ embryos were confirmed using additional marker genes, *Ebf1* for PT, *Lhx2* for TH and *Six3* for PTH (Garel et al., 1997; Nakagawa and O'Leary, 2001; Puelles et al., 2006; Fig. 3*B–E* and data not shown).

The hypothalamus can be subdivided into three main regions, the mammillary area (MAM), the tuberal hypothalamus (TUB) and the anterior hypothalamus (ANT). According to the revised prosomeric model (Puelles et al., 2012; Zhang and Alvarez-Bolado, 2016), the MAM and TUB are in the basal plate of the hypothalamus, while the ANT (also called alar hypothalamus) is in the alar plate (Fig. 3*A*). The preoptic area (POA), formerly considered as a hypothalamic region, is actually part of the telencephalon. *Nkx2.1* is expressed in the hypothalamus in response to Shh signals from the underlying mesendoderm (Dale et al., 1997; Zhao et al., 2012; Blaess et al., 2015). *Nkx2.1* is also expressed in two telencephalic structures, the POA and medial ganglionic eminence (MGE; Fig. 3*F*,*H*,*J*). In *Ftm*^−/−^ embryos, the *Nkx2.1* expression domain was preserved in the telencephalon (Fig. 3*F*,*G*,*J*,*K*) but strongly reduced in the hypothalamus (Fig. 3*H–K*). *Dbx1* expression in progenitors of the TUB (Fig. 3*L*,*M*,*P*,*Q*) and MAM (Fig. 3*N–Q*) regions was severely reduced as well, whereas it was maintained and even expanded in the thalamus (Fig. 3*L–Q*). Analysis of *Pitx2* (Fig. 3*R*,*S*) and *Wnt8b* (Fig. 3*T*,*U*) expression confirmed the reduction in the surface of the MAM in *Ftm*^−/−^ embryos. *Ebf1* expression in the ANT was still present but fused at the midline (Fig. 3*B–E*).

These data strongly suggest a severe reduction or loss of the basal plate and ventral midline of the forebrain in *Ftm*^−/−^ embryos. Conversely, the alar plate of the diencephalon appears expanded ventrally at all anteroposterior levels.

### The rostral thalamus is absent in *Ftm*^−/−^ embryos

**Figure 4.**
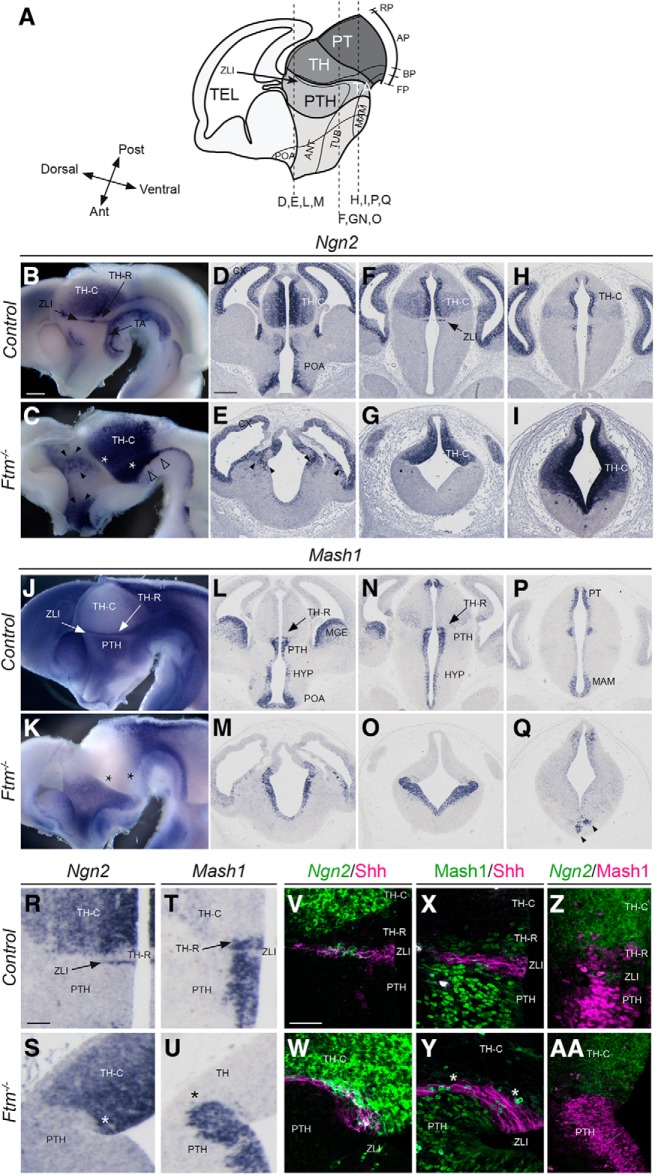
Progenitor domains at E12.5-E13.5. ***A***, Schematic drawings of the E13.5 forebrain in sagittal view. The position of the coronal sections (***D***–***I***, ***L***–***Q***) shown below is indicated with dashed lines. ***B***–***U***, ISH with probes for *Ngn2* (***B***–***I***, ***R***, ***S***) and Mash1 (***J***–***Q***, ***T***, ***U***) in whole-mount hybridization on sagittally-bisected brains viewed from the ventricular side (***B***, ***C***, ***J***, ***K***) or on coronal sections at different anteroposterior levels (***D***–***I***, ***L***–***Q***, ***R***–***U***). The genotype is indicated on the left. ***V***, ***W***, Shh immunofluorescence (magenta) combined with *Ngn2* fluorescence ISH (green). ***X***, ***Y***, Double-immunofluorescence for Shh (magenta) and Mash1 (green). ***Z***, ***AA***, Mash immunofluorescence (magenta) combined with Ngn2 fluorescence ISH (green). ***C***, ***E***, Black arrowheads point to patchy *Ngn2* expression in the prethalamus and white arrowheads point to missing *Ngn2* expression domain in the ventral forebrain. ***Q***, Black arrowheads point to remnants of the MAM. ***C***, ***K***, ***S***, ***Y***, Asterisks point to the absence of the *Ngn2*-negative, *Mash1*-positive TH-R in *Ftm*^−/−^ embryos. Ant, anterior; AP, alar plate; BP, basal plate; FP, floor plate; RP, roof plate; Post, posterior; TEL, telencephalon. Scale bars: (in ***B*** for whole-mount ISH, in ***D*** for coronal sections) ***B***–***Q***, 0.5 mm; (in ***R***) ***R***–***U***, 100 μm; (in ***V***) ***V***–***AA***, 50 μm.

We took advantage of the expression of two proneural genes, *Ngn2*, and *Mash1/Ascl1*, expressed in distinct and complementary progenitor domains (Fode et al., 2000), to analyze diencephalic subdivisions with greater precision. *Ngn2* is expressed in progenitors of most of the TH, in the ZLI and in the TAs of the diencephalon, in a domain in the POA and in the dorsal telencephalon (Fode et al., 2000; Vue et al., 2007; Fig. 4*B*,*D*,*F*,*H*). *Mash1* is expressed in progenitors of the PTH, in the prospective rostral thalamus (TH-R; see next paragraph) and in different hypothalamic subdivisions (McNay et al., 2006; Vue et al., 2007; Kim et al., 2008; Fig. 4*J*,*L*,*N*,*P*). *In Ftm*^−/−^ embryos, *Ngn2* expression was lost in the TA (empty arrowheads) and activated ectopically in a salt-and-pepper manner in regions adjacent to the telencephalon (black arrowheads), suggesting a perturbation of the telencephalic–diencephalic boundary (Fig. 4*C*,*E*,*G*,*I*). *Mash1* was still expressed in the PTH and hypothalamus (HYP; Fig. 4*K*,*M*,*O*,*Q*), but very reduced caudally (in the MAM; Fig. 4*Q*, black arrowheads). The analysis of *Ngn2* and *Mash1* expression also revealed a thickening of the progenitor domains in the TH and PTH at E12.5–E13.5 (Fig. 4*B–Q*), suggesting a delay in neurogenesis and/or an increased proliferation potential of forebrain progenitors in *Ftm*^−/−^ embryos.

The nested domains of *Ngn2* and *Mash1* expression in the diencephalon prefigure the intrinsic subdivision of the thalamus into anterior (TH-R) and posterior [caudal thalamus (TH-C)] territories (Vue et al., 2007; Fig. 4*B*,*J*). *Ngn2* and *Mash1* domains in the TH and PTH, respectively, were continuous in *Ftm*^−/−^ embryos, suggesting a perturbation of thalamic subdivisions (Fig. 4*C*,*K*, asterisks). This was confirmed by a closer examination of *Ngn2* and *Mash1* nested expression domains (Fig. 4*R–AA*). We performed combined Shh/*Ngn2* (Fig. 4*V*,*W*), Shh/Mash1 (Fig. 4*X*,*Y*) and *Ngn2*/Mash1 (Fig. 4*Z*,*AA*) fluorescence ISH and immunostaining to analyze the relationship of the different diencephalic domains with respect to the ZLI. In *Ftm*^−/−^ embryos, the domain of *Mash1* expression posterior to the Shh-positive ZLI was lost (Fig. 4*Y*, white asterisks). The *Ngn2*-positive TH-C and Mash1-positive PTH domains abutted at the level of the ZLI (Fig. 4*W*,*Y*,*AA*).

The TH-R contributes to GABAergic nuclei that participate in the subcortical visual shell, involved in the entrainment of the circadian rhythm (Delogu et al., 2012). Thus, neurons of the TH-R express *Gad67* like those of the PTH, while neurons of the TH-C do not (Fig. 2*V*). In *Ftm*^−/−^ embryos, the stripe of *Gad67* expression in the thalamus was absent, confirming the loss of the TH-R (Fig. 2*V*,*W*).

In conclusion, the TH-R is lost in *Ftm*^−/−^ embryos and the TH-C now abuts the ZLI. A diagram summarizing the *Ftm* mutant forebrain phenotype is provided in Figure 11*A*.

### Optic vesicles form in *Ftm*^−/−^ embryos and display patterning defects

Because eyes were absent in *Ftm*^−/−^ fetuses at the end of gestation (Fig. 1*I–L*), we investigated eye formation and patterning at E11.5. Eye development begins with the formation of the eye field in the alar hypothalamus and its separation into two bilaterally symmetrical optic vesicles. The expanding optic vesicles induce the surface ectoderm to form the lens placodes. The optic vesicle separates into the optic stalk proximally and the optic cup distally. Then the optic cup invaginates with the lens placode, forming two layers, the outer layer differentiates into the retinal pigmented epithelium (RPE) and the inner layer into the neural retina (Furimsky and Wallace, 2006).

**Figure 5.**
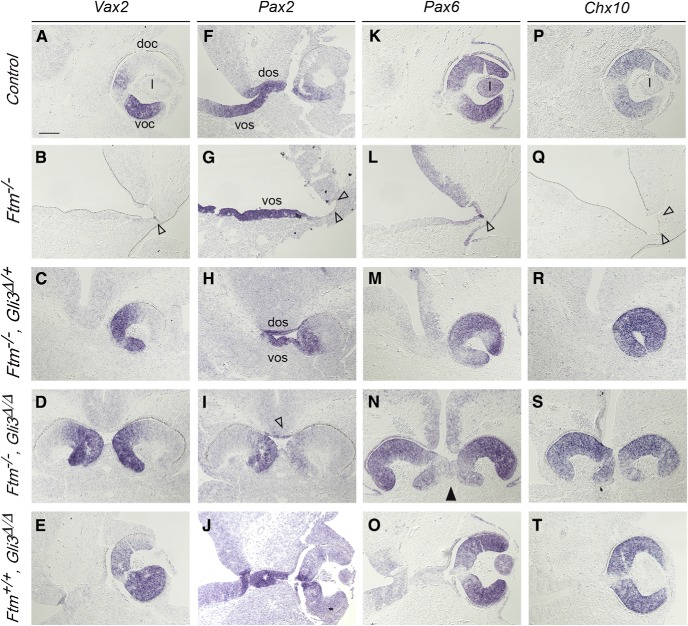
Eye morphogenesis in E11.5 embryos. ***A***–***T***, ISH on coronal sections in the region of the eye of E11.5 *Ftm*^+/+^ (***A***, ***E***, ***I***, ***M***), *Ftm*^−/−^ (***B***, ***F***, ***J***, ***N***), *Ftm*^−/−^, *Gli3*^Δ/+^ (***C***, ***G***, ***K***, ***O***), *Ftm*^−/−^, *Gli3*^Δ/Δ^ (***D***, ***H***, ***L***, ***P***), and *Ftm*^+/+^, *Gli3*^Δ/Δ^ (***E***, ***J***, ***O***, ***T***) with probes for *Vax2* (***A***–***E***), *Pax2* (***F***–***J***), *Pax6* (***K***–***O***), and *Chx10* (***P***–***T***). ***B***, ***G***, ***L***, ***Q***, Empty arrowheads point to the missing optic cup; empty arrowhead in I points to the reduced optic stalk. ***N***, Black arrowhead points to partially fused optic cups. doc, Dorsal optic cup; dos, dorsal optic stalk; l, lens; voc, ventral optic cup; vos, ventral optic stalk. Scale bars: (in ***A***) 100 μm.

**Figure 6.**
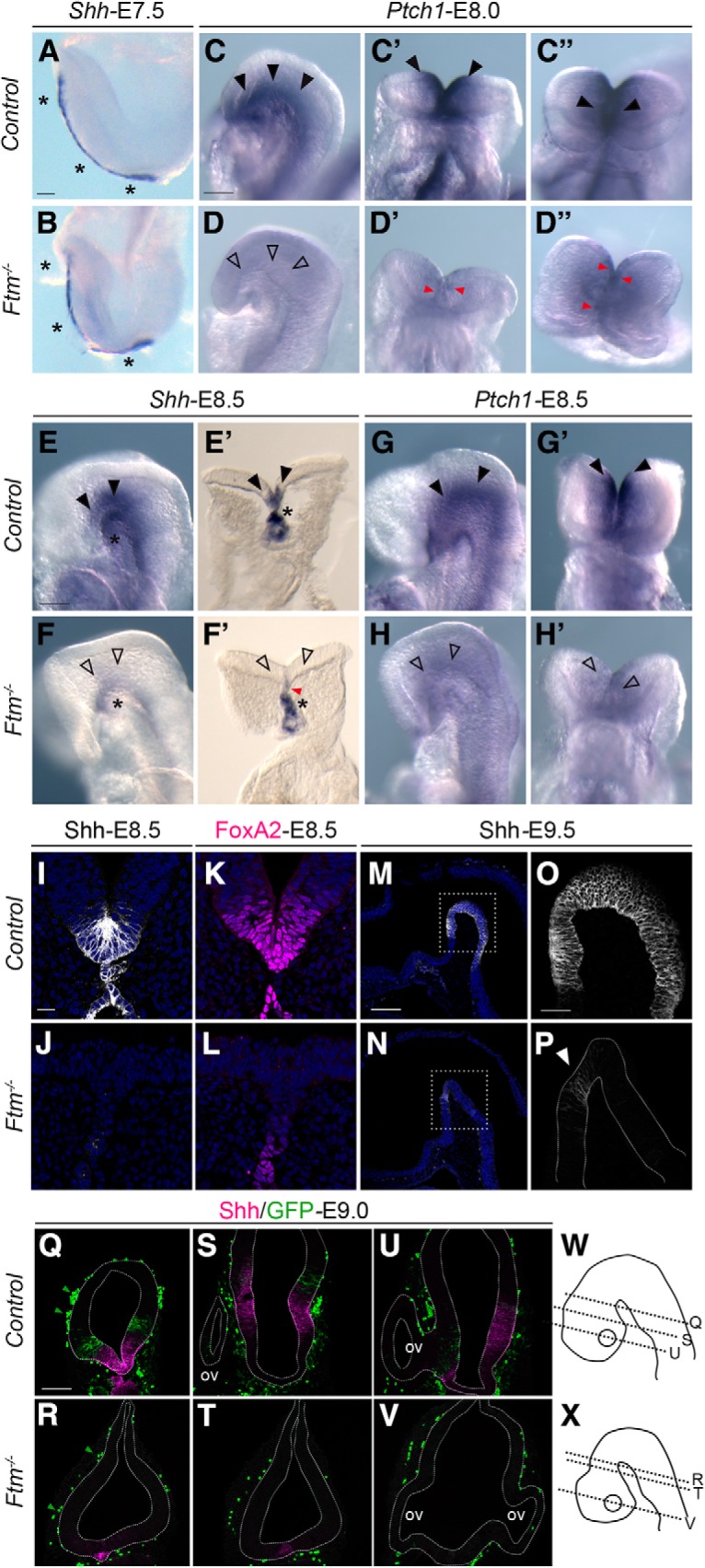
Shh expression and signaling in the E7.5-E9.5 embryo forebrain. ***A***–***H***, Whole-mount ISH on E7.5 (***A***, ***B***), E8.0 (***C***–***D″***), and E8.5 (***E***–***H′***) embryos with probes for *Shh* (***A***, ***B***, ***E***–***F′***) or *Ptch1* (***C***–***D″***, ***G***–***H″***). ***C***–***D″***, Lateral, ventral, and top views of the anterior neural plate of the same control and *Ftm*^−/−^ embryos, respectively. ***E′***, ***F′***, Vibratome sections at the level of the forebrain of embryos seen in ***E*** and ***F***, respectively. ***G***–***H′***, Lateral and ventral views of the anterior neural plate of the same control and *Ftm*^−/−^ embryos, respectively. Black arrowheads indicate *Shh* and *Ptch1* expression sites in the neural plate; black asterisks indicate *Shh* expression in the mesoderm underlying the neural plate. ***D***, ***F***, ***H***, Empty arrowheads indicate severe reduction of *Shh* and *Ptch1* expression in the neural plate of *Ftm*^−/−^ embryos. Red arrowheads point to examples of cells with remnants of *Shh* or *Ptch1* expression. ***I***–***P***, IF for Shh (***I***, ***J***, ***M***–***P***) and FoxA2 (***K***, ***L***) on coronal sections of E8.5 (***I–L***) and sagittal sections of E9.5 (***M–P***) embryos. ***P***, White arrowhead points to the small dot of Shh expression in the basal plate of *Ftm*^−/−^ embryos. ***Q***–***V***, Double IF for Shh and GFP in Tg[GBS::GFP] transgenic embryos. Green arrowheads point to GFP-positive blood cells. The genotypes are indicated on the left. Control stands for *Ftm*^+/+^ or *Ftm*^+/−^. ***W***, ***X***, Schematics indicating the approximate levels of sections in ***Q***–***V***. Note that the sections are tilted, so they do not look bilaterally symmetric. Ov, Optic vesicle. Scale bars: (in ***A***) ***A***, ***B***, (in ***C***) ***C***–***D″***, (in ***E***) ***E***–***H′***, 50 μm; (in ***I***) ***I–L***, 20 μm; (in ***M***), ***M***, ***N***, 500 μm; (in ***O***) ***O***, ***P***, (in ***Q***) ***Q***–***V***, 100 μm.

We analyzed the expression patterns of the *Pax2*, *Vax2*, *Pax6*, and *Chx10* transcription factor genes, which define distinct eye territories (Furimsky and Wallace, 2006). At this stage, *Pax6* and *Pax2* are expressed in the optic cup and optic stalk, respectively (Fig. 5*F*,*K*), where they repress each other. *Pax6* is required for optic cup formation, whereas *Pax2-null* mice display increased optic cups at the expense of optic stalk (Schwarz et al., 2000). *Chx10* is also expressed in the optic cup (Fig. 5*P*). *Vax2* is expressed in the ventral domain of the optic cup (Fig. 5*A*), where it promotes ventral optic fates. In *Ftm*^−/−^ embryos, the neural retina was absent as assessed by the absence of *Chx10* and *Vax2* expression (Fig. 5*B*,*Q*). Only a tiny region of the RPE could be detected thanks to cell pigmentation (Fig. 5*B*,*G*,*L*, empty arrowheads). *Pax2* was expressed, indicating the presence of the optic stalk (Fig. 5*G*), which suggests correct eye-field separation. Consistently, optic vesicles formed in E9 *Ftm*^−/−^ embryos as in controls (Fig. 6*S*,*U*,*V*).

In conclusion, in *Ftm*^−/−^ embryos, eye-field separation occurs correctly but proximodistal patterning of the optic vesicle is incorrect, leading to an absence of the optic cup and of lens induction.

### Shh expression and pathway activity are impaired in the forebrain of *Ftm*^−/−^ embryos

The reduction of the ventral forebrain in *Ftm* mutants suggests defects in the Hh pathway. To test this hypothesis, we analyzed Shh signaling activity in the forebrain by ISH and IF for *Shh* itself and for the Hh target genes *Ptc1*, *Gli1*, and *FoxA2*. In addition, to obtain a context-independent assay of Hh transcriptional activity through Gli transcription factors binding to their DNA targets, we introduced into the *Ftm* mutant background the Tg[GBS::GFP] reporter transgenic line in which GFP expression is driven by a concatemer of Gli-binding sites (Balaskas et al., 2012).

In mouse embryos, *Shh* is initially expressed from E7.5 in axial tissues underlying the neural plate (notochord posteriorly and prechordal plate anteriorly), where it signals to the overlying neural plate to induce ventral structures. Shh signaling induces *Shh* expression in the ventral forebrain from E8.0 onward (Dale et al., 1997). Whereas *Shh* expression in the axial mesoderm was unperturbed in E7.5 *Ftm*^−/−^ embryos compared with controls (Fig. 6*A*,*B*, asterisks), *Ptch1* expression in the ventral neural plate (Fig. 6*C–C″*, black arrowheads) was reduced in *Ftm*^−/−^ (Fig. 6*D–D″*, empty arrowheads) with few remaining *Ptch1*-positive cells (Fig. 6*D–D″*, red arrowheads). In E8.5 control embryos, *Shh* is still expressed in the mesendoderm underlying the brain (Fig. 6*E*,*E′*, asterisks, *I*). In addition, it is activated in the ventral neural tube, including the ventral forebrain (Dale et al., 1997; Alvarez-Bolado et al., 2012; Fig. 6*E*,*E′*, black arrowheads, *I*). In E8.5 *Ftm*^−/−^ embryos, *Shh* expression persisted in the notochord and prechordal plate (Fig. 6*F*,*F′*,*J*, black asterisks) but was severely downregulated in the ventral neural tube and brain (empty arrowheads) with few remaining positive cells (red arrowhead). *Ptch1* expression in two stripes surrounding the *Shh* expression domain in the ventral neural tube and brain of control embryos (Fig. 6*G*,*G′*, black arrowheads) was also downregulated in *Ftm*^−/−^ (Fig. 6*H*,*H′*, empty arrowheads), consistent with the reduction of *Shh* expression. *FoxA2*, a target of Shh signaling expressed in the ventral floor plate and in the ventral forebrain (Hallonet et al., 2002; Ribes et al., 2010; Fig. 6*K*), was faintly expressed in the neural plate of *Ftm*^−/−^ embryos, confirming the very low Hh/Gli activity (Fig. 6*L*).

At E9.0–E9.5, *Shh* expression is still present in the ventral diencephalon (Fig. 6*M*,*O*,*Q*) but in the hypothalamus it is downregulated in the most ventral region and activated in two lateral stripes in the basal plate (Fig. 6*S*,*U*; Szabó et al., 2009a; Alvarez-Bolado et al., 2012, Blaess et al., 2015). *Shh* expression was lost in the whole ventral forebrain of *Ftm*^−/−^ embryos, except in a tiny spot in the diencephalic ventral midline located approximately at the anteroposterior (AP) level of the future ZLI (Fig. 6*N*,*P*,*R*,*T*,*V*). Gli activity assessed by Tg[GBS::GFP] was observed lateral to Shh expression domain in all forebrain regions of control embryos (Fig. 6*Q*,*S*,*U*), whereas in *Ftm*^−/−^ embryos, Gli activity could not be detected (Fig. 6*R*,*T*,*V*). Note that GFP is also expressed in blood cells and that this expression was still present in *Ftm*^−/−^ embryos (Balaskas et al., 2012; Fig. 6*Q*,*R*, green arrowheads).

Overall our results show that Shh signaling activity is drastically reduced but not totally abolished in the ventral forebrain of *Ftm*^−/−^ embryos as early as E8.0.

### *Shh* expression and Hh/Gli pathway activity show different perturbations in distinct domains of the E12.5 diencephalon

**Figure 7.**
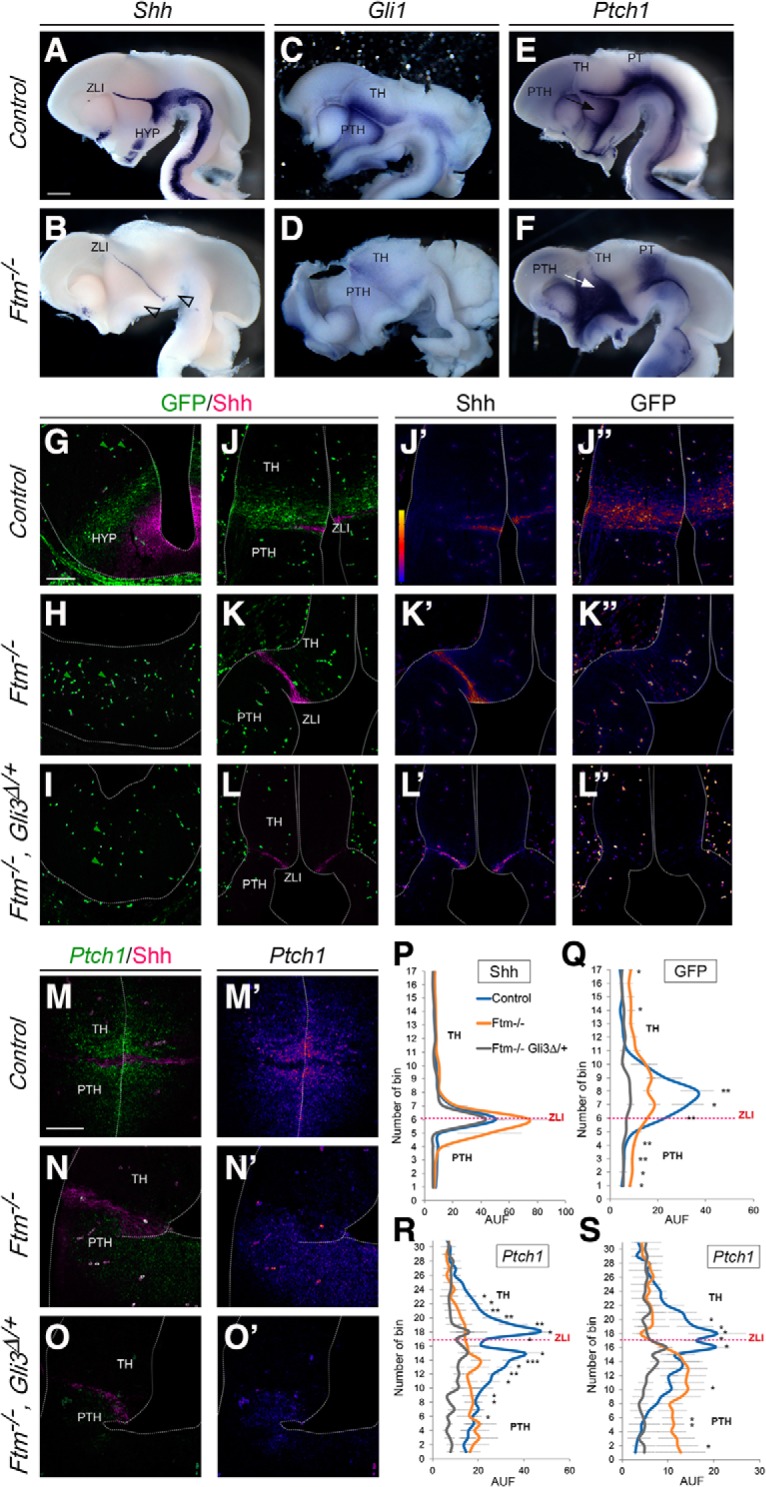
Shh expression and signaling in the E12.5 embryo forebrain. ***A***–***F***, Whole-mount ISH on E12.5 control (***A***, ***C***, ***E***) or *Ftm*^−/−^ (***B***, ***D***, ***F***) half-brains viewed from the ventricular surface, with probes for *Shh* (***A***, ***B***), *Gli1* (***C***, ***D***), or *Ptch1* (***E***, ***F***). ***G***–***L″***, IF on coronal sections of control (***G***, ***J***, ***J′***, ***J″***), *Ftm*^−/−^ (***H***, ***K***, ***K′***, ***K″***) or [*Ftm, Gli3*^Δ/+^] (***I***, ***L***, ***L′***, ***L″***) Tg[GBS::GFP] embryos. IF was performed with antibodies for Shh and GFP. ***J′***, ***J″***, ***K′***, ***K″***, ***L′***, ***L″***, Fire versions of Shh and GFP are shown (***J′***, fire scale). ***B***, Empty arrowheads point to the missing Shh expression domain in the ventral forebrain. ***M***–***O′***, Combined fluorescence ISH *Ptch1* and IF for Shh on coronal sections for of the diencephalon of E12.5 control (M, M′), *Ftm*^−/−^ (***N***, ***N′***) and compound [*Ftm*^−/−^, *Gli3*^Δ/+^] (***O***, ***O′***) embryos. ***M′***–***O′***, Fire versions of *Ptch1* FISH. ***M***–***O***, Green arrowheads point to GFP-positive blood cells. ***P***–***S***, Diagrams showing the quantification of the intensity of Shh (***P***) or GFP (***Q***) IF and *Ptch1 FISH* (***R***, ***S***) along the diencephalon. *Ptch1* FISH intensity was quantified next to the ventricular surface (***R***) or 40 μm away from the ventricular surface (***S***). Numbers on the abscissa relate to the position of the squares of quantification. Fluorescence intensity in ordinate is given in arbitrary units (AUF). ***Q***–***S***, P values of statistical tests are shown as *p < 0.1, **p < 0.01, and ***p < 0.001. No asterisk means that the difference was found nonsignificant by the statistical test. Scale bars: (in ***A***) ***A***–***F***, 0.5 mm; (in ***G***) ***G***–***O′***, 100 μm.

We next investigated Hh/Gli pathway activity at E12.5, when the ZLI is fully formed and secretes Shh to organize cell fate in the thalamus and prethalamus (Epstein, 2012; Zhang and Alvarez-Bolado, 2016). The ZLI was formed in both control and *Ftm*^−/−^ embryos, and its DV extent was increased in *Ftm*^−/−^ compared with control embryos (Fig. 7*A*,*B*). In contrast, *Shh* expression was absent from the ventral forebrain of *Ftm*^−/−^ embryos (Fig. 7*B*, empty arrowheads). To test whether Shh signaling was active at the ZLI, we analyzed *Ptch1* and *Gli1* expression as well as Gli transcriptional activity with Tg[GBS::GFP]. Surprisingly, *Gli1* and *Ptch1* were differently affected in *Ftm*^−/−^ embryos (Fig. 7*C–F*). *Gli1* expression was dampened in the regions close to the *Shh* expression domains (Fig. 7*C*,*D*). In contrast, *Ptch1* expression was totally downregulated in the thalamus and upregulated in the prethalamus and pretectum (Fig. 7*E*,*F*). Using FISH on sections and signal quantification, we confirmed the differential expression of *Ptch1* on both sides of the ZLI in *Ftm*^−/−^ embryos and found that this upregulation was more striking at a distance from the ventricular surface (Fig. 7*M–N′*,*R*,*S*). To test whether this reflected differential Gli activity on both sides of the ZLI, we observed GFP expression in Tg[GBS::GFP] embryos. At this stage, GFP-positive blood cells were present within the neural tube in all genotypes examined (Fig. 7*G–I*, green arrowheads point to examples of these GFP-positive blood cells). We found that Gli activity was downregulated in the diencephalon and hypothalamus of *Ftm*^−/−^ embryos compared with controls, in the ventral regions (Fig. 7*G*,*H*) as well as on both sides of the ZLI (Fig. 7*J–K″*). However, Gli activity was not totally absent on both sides of the ZLI (Fig. 7*K–K″*), as confirmed by quantification of fluorescence intensity (Fig. 7*Q*). Moreover, in *Ftm*^−/−^ embryos, the Shh-positive ZLI was wider along the AP axis [Fig. 7*J–K′*,*M*,*N*,*P*; width of the ZLI measured at the ventricular surface: 53 ± 11 μm for controls (*n* = 6) and 109 ± 26 μm for *Ftm*^−/−^ (*n* = 4); *p* = 0.0095].

In conclusion, in the *Ftm*^−/−^ embryos, *Shh* expression is strongly reduced in the ventral forebrain but maintained and even expanded in the ZLI. Gli activity is dampened in regions adjacent to Shh-expressing domains. The loss of Ftm also uncovered a differential prepattern of *Ptch1* and *Gli1* expression in different diencephalic prosomeres.

### Reintroduction of Gli3R into the *Ftm* background rescues aspects of the forebrain phenotype

The impaired production of Gli3R in *Ftm*^−/−^ embryos (Vierkotten et al., 2007; Besse et al., 2011) could participate in the observed phenotype. We thus tested how Gli activity in the diencephalon was modified in compound [*Ftm*^−/−^; *Gli3*^Δ/+^] embryos, by performing quantification of *GFP* and *Shh* expression in [*Ftm*^−/−^*; Gli3*^Δ/+^] mutant embryos harboring Tg[GBS::GFP]. The *Gli3*^Δ*699*^ allele produces constitutively a short form of Gli3 with partial repressor activity (Hill et al., 2007; Cao et al., 2013). We found that the Shh-dependent Gli activity adjacent to the ZLI was reduced in these compound mutants (Fig. 7*I*, *L–L″*,*Q*). *Ptch1* expression in the prethalamus was also downregulated (Fig. 7*O*,*O′*,*R*,*S*). Moreover, the increased width of *Shh* expression in the ZLI was rescued in double mutants [Fig. 7*J–P*; width of the ZLI measured at the ventricular surface: 109 ± 26 μm for *Ftm*^−/−^ (*n* = 4) and 50 ± 12 μm for (*Ftm*^−/−^ *Gli3*^Δ/+^); *p* = 0.028].

**Figure 8.**
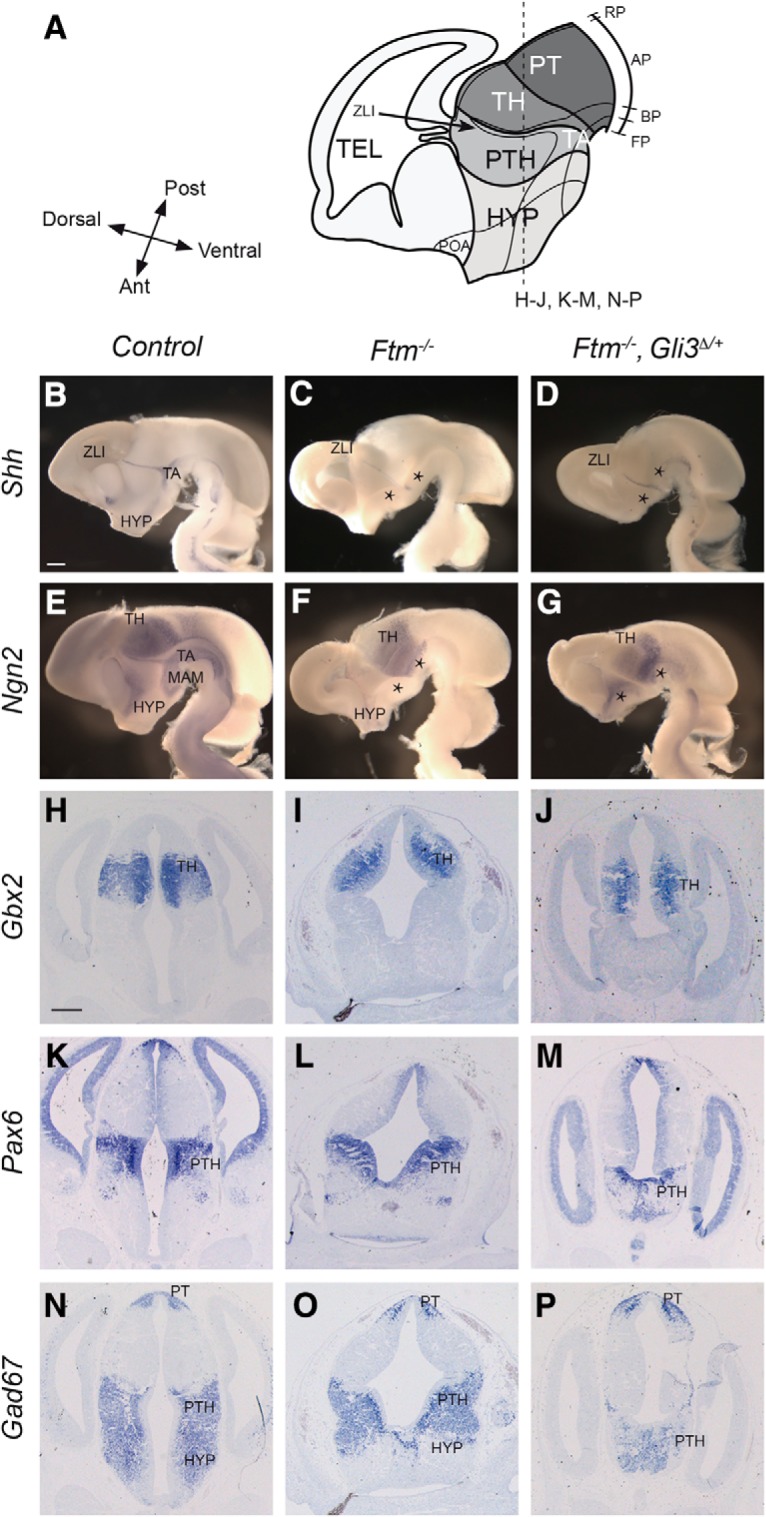
Diencephalon and hypothalamus patterning in compound [*Ftm, Gli3*^Δ*699*^] mutants. ***A***, Schematic drawings of the E13.5 forebrain in sagittal view. The position of the coronal sections (***H***–***P***) shown below is indicated with dashed lines. ***B***–***G***, Whole-mount ISH with probes for *Shh* (***B***–***D***) or *Ngn2* (***E***–***G***) on sagittally-bisected brains viewed from the ventricular side. ***C***, ***D***, ***F***, ***G***, Black asterisks point to the absence of ventral forebrain. ***H***–***P***, ISH on coronal sections with probe for *Gbx2* (***H***–***J***), *Pax6* (***K***–***M***), or *Gad67* (***N***–***P***). The genotype of the embryo is indicated at the top. Ant, Anterior; AP, alar plate; BP, basal plate; FP, floor plate; RP, roof plate; Post, posterior; TEL: telencephalon. Scale bars: (in ***B*** for coronal sections, in ***H*** for whole-mount ISH), 0.5 mm.

We then tested the consequences of Gli3R reintroduction on forebrain patterning and eye formation. ISH for *Shh*, *Ngn2*, *Gbx2*, *Pax6*, and *Gad67* indicated that the reduction of the ventral forebrain was still observed and even worsened in [*Ftm*^−/−;^ *Gli3*^Δ/+^] (Fig. 8*B–P*). As in *Ftm*^−/−^ embryos, the alar plate of the diencephalon was expanded ventrally (Fig. 8*E–P*), and *Shh* expression was absent from the ventral forebrain but present in the ZLI (Fig. 8*C*,*D*). In contrast, optic cup formation was restored in compound mutants, and the optic cup showed correct DV patterning (Fig. 5*C*,*D*,*H*,*I*,*M*,*N*,*R*,*S*). However, the eyes were internalized and brought together in [*Ftm*^−/−^*; Gli3*^Δ/+^] embryos, and even more in [*Ftm*^−/−^*; Gli3*^Δ/Δ^] embryos, and this was associated with a very reduced optic stalk (Fig. 5*C*,*D*,*H*,*I*,*M*,*N*,*R*,*S*). We also analyzed [*Ftm*^+/+^; *Gli3*^Δ/Δ^] embryos, which looked similar to controls (Fig. 5*E*,*J*,*O*,*T*) as found in another study (Christoph Gerhard, University of Düsseldorf, personal communication), indicating that only GliR is required for optic cup formation.

In conclusion, reintroducing Gli3R into the *Ftm* background rescues some of the defects of *Ftm*^−/−^ embryos, such as optic cup agenesis and ZLI enlargement, but not others such as the reduction of the forebrain basal plate and of the rostral thalamus. Moreover, it triggers optic stalk hypoplasia.

### The Wnt/β-catenin pathway appears unperturbed in the *Ftm*^−/−^ embryonic diencephalon

**Figure 9.**
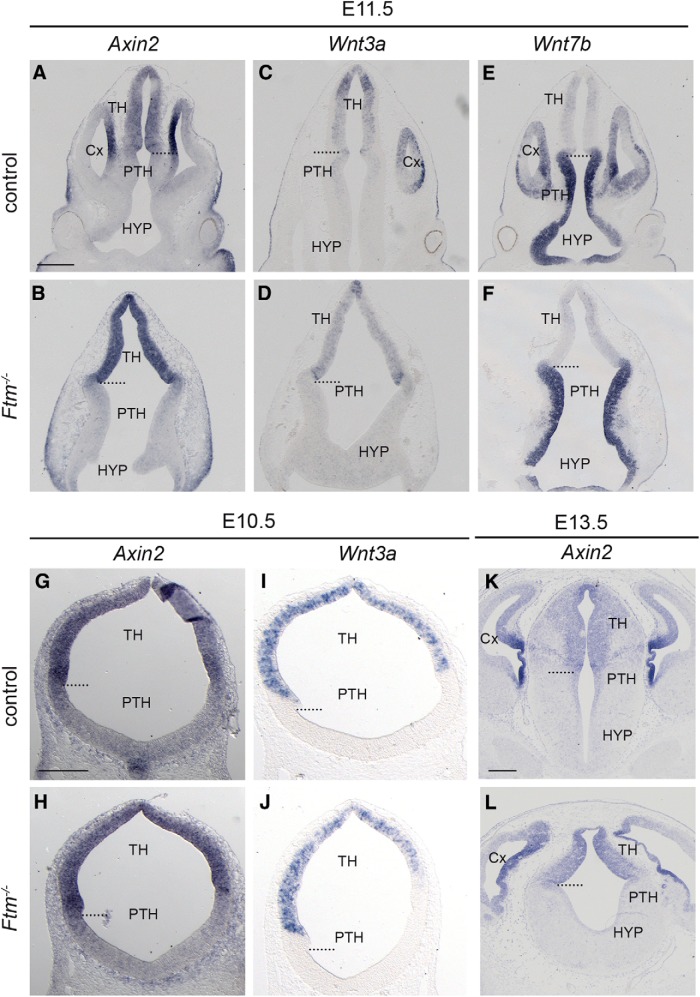
Wnt/β-catenin pathway activity in the forebrain of *Ftm* mutants. ISH with probes for *Axin2* (***A***, ***B***, ***G***, ***H***, ***K***, ***L***), *Wnt3a* (***C***, ***D***, ***I***, ***J***), and *Wnt7b* (***E***, ***F***) on coronal sections of E11.5 (***A***–***F***), E10.5 (***G***–***J***), and E13.5 (***K***, ***L***) embryos at the level of the forebrain. The genotype is indicated on the left of the Figure. The dotted lines indicate the position of the ZLI. Scale bars: (in ***A***) ***A***–***F***, (in ***G***) ***G***–***J***, (in ***K***) ***K***, ***L***, 0.5 mm.

Perturbations in the Wnt/β-catenin pathway have been observed in ciliary gene mutants but their extent and nature depend on the tissue and gene analyzed (Simons et al., 2005; Ocbina et al., 2009; Lancaster et al., 2011). Wnt/β-catenin signaling in the diencephalon is involved in specifying thalamic identity and later in promoting formation of the TH-C at the expense of TH-R, in parallel to (and independently of) Shh signaling (Braun et al., 2003; Zhou et al., 2004; Bluske et al., 2012). We thus tested whether the activity of the Wnt/β-catenin pathway was perturbed in the diencephalon of *Ftm*^−/−^ embryos, using *Axin2* as a target of the pathway (Bluske et al., 2009). We also examined the expression of *Wnt3a* and *Wnt7b*, two *Wnt* genes expressed in the developing diencephalon (Bluske et al., 2009). The expression of *Axin2* (Fig. 9*A*,*B*,*G*,*H*,*K*,*L*), *Wnt3a* (Fig. 9*C*,*D*,*I*,*J*), and *Wnt7b* (Fig. 9*E*,*F*) was similar in control and *Ftm*^−/−^ embryos from E10.5 to E13.5, strongly suggesting that the Wnt/β-catenin pathway is not perturbed in the *Ftm*^−/−^ diencephalon.

### Cilia of forebrain neural progenitors are severely reduced in number and malformed in *Ftm*^−/−^ embryos

**Figure 10.**
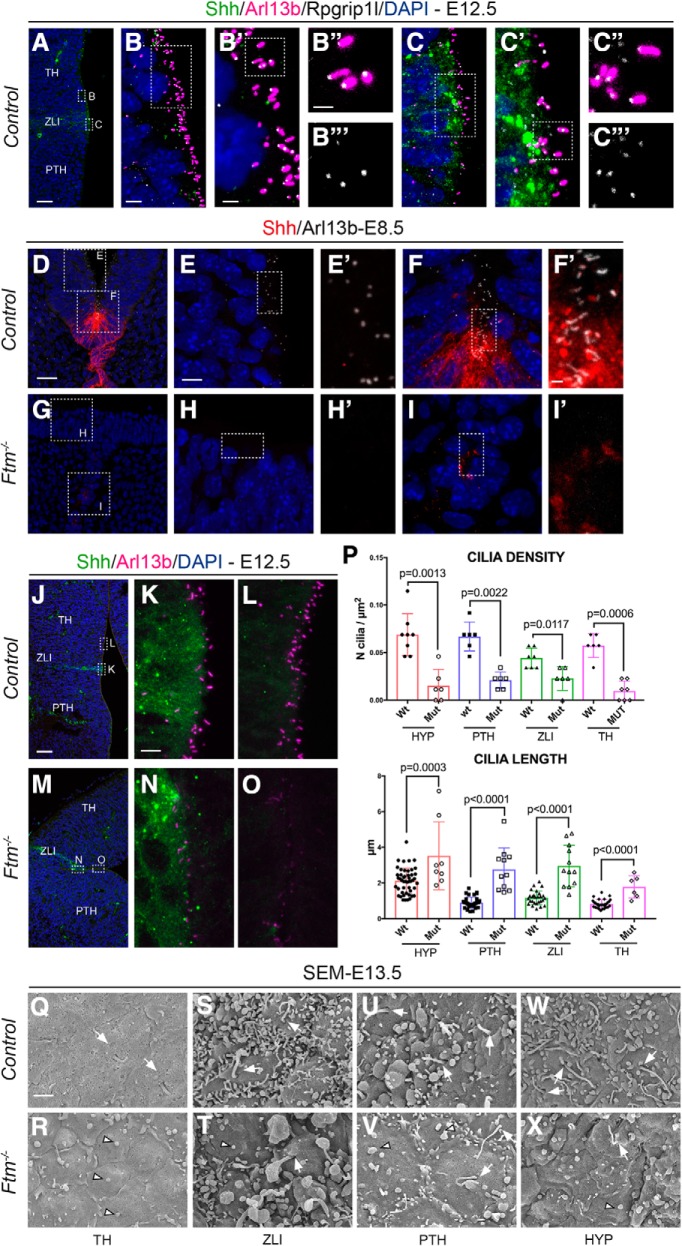
Cilia in the forebrain of *Ftm* mutants. ***A***–***C‴***, Immunofluorescence on coronal sections of E12.5 control embryos with antibodies for Shh (green), Arl13b (magenta), and Rpgrip1l (white). Nuclei are stained with DAPI. ***B‴***, ***C‴***, Only Rpgrip1l is shown. ***D***–***I′***, Immunofluorescence on coronal sections of E8.5 control (***D***–***F′***) and *Ftm*^−/−^ (***G***–***I′***) embryos with antibodies for Shh (red) and Arl13b (white). Nuclei are stained with DAPI. ***D***, ***G***, White squares indicate the regions magnified in ***E***, ***F***, and ***H***, ***I***, respectively. ***E***–***I***, White rectangles indicate the regions magnified in ***E′***–***I′***, respectively. ***J***–***O***, Immunofluorescence on coronal sections of E12.5 control (***J***–***L***) and *Ftm*^−/−^ (***M***–***O***) embryos with antibodies for Shh (green) and Arl13b (magenta). ***J***, ***M***, Nuclei are stained with DAPI. White rectangles indicate the regions magnified in ***K***, ***L*** and ***N***, ***O***, respectively. ***P***, Graph comparing the density (top) and length (bottom) of cilia on the SEM images, in the HYP, PTH, TH, and ZLI regions of control and *Ftm*^−/−^ embryos. ***Q***–***X***, SEM of the ventricular surface in different regions of control (***Q***, ***S***, ***U***, ***W***) and *Ftm*^−/−^ (***R***, ***T***, ***V***, ***X***) hemisected brains. White arrows point to the base of cilia, white arrowheads point to button-like structures surrounded by a ciliary pocket, similar to those found in the cortex of *Ftm*^−/−^ embryos (Besse et al., 2011). Scale bars: (in ***A***, ***J***) ***A***, ***J***, ***M***, 50 μm; (in ***D***) ***D***, ***G***, 20 μm; (in ***B***) ***B***, ***C***, (in ***E***) ***E***, ***F***, ***H***, ***I***, 5 μm; (in ***B′***) ***B′***, ***C′***, (in ***K***) ***K***, ***L***, ***N***, ***O***, 2 μm; (in ***B″***) ***B″***, ***B‴***, ***C″***, ***C‴***, (in ***F′***) ***F′***, ***G′***, ***H***, ***I′***, (in ***Q***) ***Q***–***X***, 1 μm.

In the telencephalon of *Ftm*^−/−^ embryos, neural progenitors are devoid of primary cilia (Besse et al., 2011). Because our data indicate that Shh signaling activity is not totally lost in the diencephalon and hypothalamus of *Ftm*^−/−^ embryos, we tested the status of cilia in this region. We first analyzed Rpgrip1l expression in E12.5 controls and found that it was present at the ciliary transition zone in different diencephalic domains including the ZLI (Fig. 10*A–C‴*). We then compared cilia in the control and *Ftm* mutant brain at different stages by immunofluorescence for Arl13b. Arl13b-positive cilia were present in the forebrain of E8.5 control embryos (Fig. 10*D–F′*) but were not detected in *Ftm*^−/−^ embryos (Fig. 10*G–I′*). In the E12.5 diencephalon, Arl13b-positive cilia were present in the TH, PTH and ZLI in control embryos (Fig. 10*J–L*) and severely reduced in number in *Ftm*^−/−^ embryos (Fig. 10*M–O*). Arl13b staining was less intense in the remaining cilia (Fig. 10*M–O*), suggesting that the number of cilia in *Ftm* mutants might be underestimated using Arl13b as a marker. Indeed, Rpgrip1l is required for Arl13b ciliary localization in several cell types (Wiegering et al., 2018). To further investigate cilium number and shape, we performed scanning electron microscopy (SEM) of the ventricular surface of E13.5 control and *Ftm*^−/−^ brains, at different AP levels: in the TH, ZLI, PTH, and HYP (Fig. 10*Q–X*). In control embryos, cilia of ∼1 μm in length were found in the TH, PTH, and ZLI (Fig. 10*P*,*Q*,*S*,*U*, arrows), whereas in the HYP cilia were in average 2 μm long (Fig. 10*P*,*W*, arrows). Cilia were more difficult to recognize in the ZLI because the ventricular surface of the cells was rich in protrusions and vesicles (Fig. 10*S*). In the diencephalon and hypothalamus of the *Ftm*^−/−^ forebrain, cilia were in majority absent or reduced to button-like structures (Fig. 10*R*,*T*,*V*,*X*, arrowheads), with a few very long cilia often abnormal in shape (Fig. 10*R*,*T*,*V*,*X*, arrows). These remaining cilia were present in all regions, but more frequently in the ZLI (Fig. 10*T*).

In conclusion, cilia were absent from the forebrain of *Ftm*^−/−^ embryos as soon as E8.5. At E12.5 they were reduced in number in the diencephalon and hypothalamus, and the remaining cilia were longer than in controls and often presented an abnormal shape.

## Discussion

**Figure 11.**
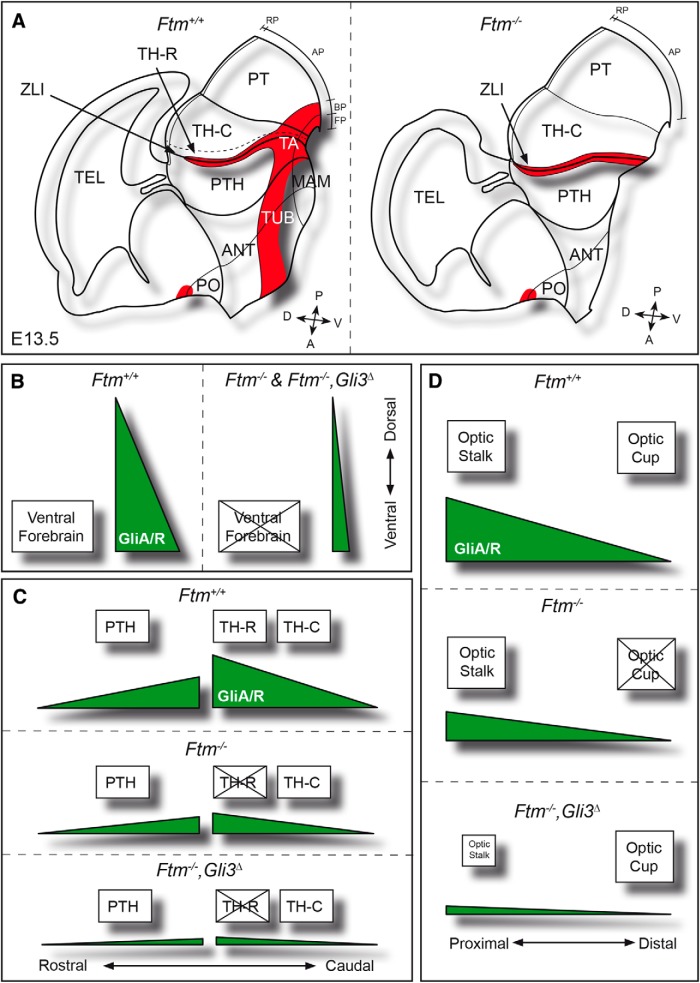
Schematics of forebrain patterning defects in *Ftm* embryos and their link to perturbations of Gli activity. ***A***, Schematic drawings of the forebrain of E13.5 control (left) and *Ftm*^−/−^ (right) embryos. Shh expression domains are in red. ***B***–***D***, Interpretive schematics of the GliA/GliR ratios (green) during ventral forebrain formation (***B***), alar diencephalon patterning (***C***), and optic vesicle patterning into optic stalk and optic cup (***D***) in control, *Ftm*^−/−^ and [*Ftm*^−/−^, *Gli3*^Δ^] embryos. ***B***, From E8.0 onward, a high GliA/GliR ratio is required for the formation of the ventral forebrain. In *Ftm*^−/−^ as well as in compound [*Ftm*^−/−^, *Gli3*^Δ^] embryos, the reduction this ratio causes a strong reduction of the ventral forebrain. ***C***, At later stages (E10.5–12.5), in the alar diencephalon, a high GliA/GliR ratio is required for TH-R formation, whereas a lower ratio is sufficient for PTH and TH-C formation. In *Ftm*^−/−^ embryos the TH-R is lost but the ratio is sufficient for PTH and TH-C formation. ***D***, From E9.0 onward, in the optic vesicle, optic stalk formation requires a high GliA/GliR ratio, while the optic cup requires that only GliR is present. Low levels of GliA are sufficient for optic stalk formation in *Ftm*^−/−^ embryos. In contrast, the optic cup is not formed because of the reduction of GliR levels. In compound [*Ftm*^−/−^, *Gli3*^Δ^] embryos, the optic cup is rescued and the optic stalk is reduced (the eyes are closer to one another) because of the reintroduction of Gli3R. A, Anterior; AP, alar plate; BP, basal plate; D, dorsal; FP, floor plate; P, posterior; PO, preoptic area; RP, roof plate; TEL, telencephalon; V, ventral.

The role of cilia in the forebrain has been little studied outside of the telencephalon. In this paper we have studied the role of the *Ftm/Rpgrip1l* ciliopathy gene in patterning of the diencephalon, hypothalamus, and eyes. At the end of gestation, *Ftm*^−/−^ fetuses displayed anophthalmia, reduction of the ventral hypothalamus and disorganization of diencephalic nuclei and axonal tracts. We examined the developmental defects underlying this phenotype. *Ftm*^−/−^ embryos showed a severe reduction of ventral forebrain structures accompanied by a dorsoventral expansion of alar diencephalic domains and a loss of the rostral thalamus (Fig. 11*A*). Optic vesicles formed but optic cup morphogenesis did not occur. Investigating the molecular mechanisms of these defects, we uncovered region-specific perturbations of the Hh/Gli pathway, whereas the Wnt/β-catenin pathway appeared unaltered. Combined with our previous studies (Besse et al., 2011; Laclef et al., 2015), our data lead to a global understanding of the role of primary cilia in forebrain patterning and morphogenesis of their relationship with Shh signaling.

Do the forebrain defects of *Ftm* mutants correspond to a ciliary phenotype? Apart from the disorganization of the diencephalic-telencephalic boundary and TCA tracts (Willaredt et al., 2008, 2013; Magnani et al., 2015), the defects observed in this study have not been reported in other ciliary mutants, some of which die too early (Gorivodsky et al., 2009). It was thus important to study the number and integrity of cilia in the diencephalon and hypothalamus of *Ftm*^−/−^ embryos at different stages. We found a near-total loss of cilia in the progenitors of the forebrain of *Ftm*^−/−^ embryos at E8.5. At E12.5, cilia were severely reduced in number, and their shape and content were highly abnormal. This, combined with our previous studies (Besse et al., 2011), strongly suggests that the forebrain defects observed in *Ftm* mutants are because of the ciliary defects in neural progenitors.

Our data point to region-specific defects in Shh signaling in the forebrain of *Ftm* mutants. The reduction in ventral forebrain areas and the loss of the TH-R in *Ftm* mutants suggest an impaired response to Shh signals, similar to what has been previously observed in the ventral spinal cord of these mutants (Vierkotten et al., 2007). Indeed, tegmental areas of the diencephalon and hypothalamus depend on Shh signaling from the notochord and prechordal plate as soon as E7.5, which induces *Shh* expression in the forebrain midline. Neural Shh is in turn required from E8.5 onward for correct formation of the basal diencephalon and hypothalamus (Dale et al., 1997; Szabó et al., 2009a,b; Shimogori et al., 2010; Zhao et al., 2012). Later, at E10.5–E12.5, high Hh/Gli activity (from the ZLI and the ventral forebrain) is required for the formation of the TH-R, whereas the TH-C requires lower Hh/Gli activity (Hashimoto-Torii et al., 2003; Jeong et al., 2011). Thus, our observation of the loss of the TH-R and the expansion of the TH-C in *Ftm*^−/−^ embryos is totally consistent with the strong reduction of GliA activity as assayed by Tg[GBS::GFP] and the near-total absence of *Shh* expression in the ventral diencephalon at E12.5.

However, the phenotype of the *Ftm* mutant in the forebrain differs from that of a *Shh* mutant. In *Shh*^−/−^ embryos, unlike in *Ftm*^−/−^ embryos, the whole diencephalon is extremely reduced in size because of reduced proliferation and survival as soon as the 15 s stage (Ishibashi and McMahon, 2002). Moreover, contrary to *Shh* mutants (Chiang et al., 1996), *Ftm* mutants never show cyclopia, even when two copies of Gli3R are reintroduced. This suggests that a low level of Hh/Gli pathway activity (undetected by the GBS::GFP transgene) sufficient to separate the eye fields and to promote forebrain morphogenesis is produced from the underlying prechordal mesendoderm of *Ftm*^−/−^ embryos. Our observation of sparse *Ptch1*- and *Shh*-positive cells in the neural plate of E8.0–E8.5 mutant embryos consolidates this assumption.

In that respect, examination of Hh/Gli activity at the ZLI is very informative. Indeed, the ZLI forms in *Ftm*^−/−^ embryos and is even wider than in controls. This widening is accounted for by the reduction in Gli3R levels, because it is rescued in compound [*Ftm*^−/−^, *Gli3*^Δ/+^] embryos. Consistent with these data, Gli3 repression by Wnt signals is required for controlling the width of the ZLI in chicken embryos (Martinez-Ferre et al., 2013). Moreover, *Shh* from the ZLI appears to be able to signal, although with lower efficiency than in controls. Thus, the Hh/Gli pathway is still active in *Ftm*^−/−^ embryos.

The ZLI has been proposed, initially in chick, to form through an inductive process requiring Shh signaling from the diencephalic basal plate (Kiecker and Lumsden, 2004; Zeltser, 2005; Epstein, 2012). In mouse mutants in which expression of a functional Shh is absent from the ventral diencephalon, the ZLI does not form (Szabó et al., 2009b). If ZLI formation requires Shh signals from the basal plate, how can it occur in *Ftm* mutants, which display no *Shh* expression in the basal diencephalon? In E9.5 *Ftm*^−/−^ embryos, a discrete patch of *Shh* expression remained in the basal plate at the level of the future ZLI. We propose that this patch of *Shh* expression is sufficient for the initiation of ZLI formation in *Ftm* mutants. Because this patch does not give rise to detectable Gli activity, we speculate that here Shh might signal through Gli-independent, non-canonical pathway (Carballo et al., 2018). Alternatively, remaining cilia in the prospective ZLI could be still functional and could lead to a low level of Gli activity (undetected in our study) sufficient to initiate ZLI formation.

Examination of the eye in *Ftm* mutants provides another example of the region-specific functions of cilia. In *Ftm*^−/−^ embryos, the optic cup and lens are totally absent. Gli3 is known to be involved in optic cup formation (Furimsky and Wallace, 2006), but it was not known so far whether it acted as a repressor or as an activator. We found optic cups with correct DV patterning in compound [*Ftm*^−/−^, *Gli3*^Δ/+^] and [*Ftm*^−/−^, *Gli3*^Δ/Δ^] embryos, showing that Gli3R, and not Gli3A, is crucial for optic cup formation, and that the function of cilia in this process is mediated by Gli3R. This was confirmed by the analysis of [*Ftm*^+/+^, *Gli3*^Δ/Δ^] siblings, which displayed normal retina. The retinal phenotype of *Ftm* mutants is reminiscent to that of the telencephalon, where dorsal structures are reduced because of the reduction in Gli3R levels (Besse et al., 2011; Laclef et al., 2015). However, in compound [*Ftm*^−/−^, *Gli3*^Δ/+^] and [*Ftm*^−/−^, *Gli3*^Δ/Δ^] embryos, the optic cups were closer to each other under the ventral forebrain and even partially fused in some cases, and the optic stalk was almost totally absent. Thus, cilia are required both for GliR-dependent optic cup formation and for GliA-dependent optic stalk morphogenesis.

In conclusion, our data show that, in *Ftm* mutants, forebrain structures requiring high GliA activity, such as the rostral thalamus and ventral forebrain, and structures that require high GliR activity, such as the optic cup, are lost. In contrast, structures that require low or intermediate Hh/Gli activity, such as TH-C or the optic stalk, are still present. Thus, different regions of the forebrain are differently affected by the loss of cilia depending on their specific requirement for GliA or GliR activity (Fig. 11*B–D*).

Are our data relevant for human disease? There are few reports of hypothalamic or diencephalic malformations in ciliopathies. However, a precise analysis of the forebrain is rarely possible in fetuses with severe ciliopathies such as Meckel syndrome. Microphthalmia and benign tumors called diencephalic or hypothalamic hamartomas have been observed in Meckel syndrome and other ciliopathies (Ahdab-Barmada and Claassen, 1990; Roume et al., 1998; Paetau et al., 2008; Poretti et al., 2011, 2017; Del Giudice et al., 2014). Interestingly, diencephalic hamartomas have been linked to mutations in *GLI3* and other SHH pathway genes (Shin et al., 1999; Hildebrand et al., 2016), suggesting that those observed in ciliopathies could also be caused by defects in SHH signaling. Holoprosencephaly is rarely described in ciliopathies, and only in the most severe form, Meckel syndrome (Paetau et al., 1985, Ahdab-Barmada and Claassen, 1990). This may be surprising, given the essential role of cilia in vertebrate Shh signaling. Our study of the forebrain of *Ftm* mutants provides a potential explanation, as we find clear phenotypic differences between the *Ftm* mutants and Hh/Gli pathway mutants. Nevertheless, ciliopathy genes seem to play a role as modifier genes for HPE. HPE shows high phenotypic variability in single families, which has led to the proposal that a combination of mutations in HPE genes could account for the variable severity of the phenotype (the multi-hit hypothesis). In favor of this hypothesis, digenic or oligogenic inheritance has been identified in several HPE families (Mouden et al., 2016; Kim et al., 2019). Interestingly, homozygous mutations in the *STIL* gene encoding a pericentriolar and centrosomal protein have been found in patients with HPE and microcephaly (Mouden et al., 2015, Kakar et al., 2015). Mouse *Stil*^−/−^ embryos display severe forebrain midline defects (Izraeli et al., 1999) and ciliogenesis, centriole duplication and Shh signaling are defective in the absence of STIL (David et al., 2014; Mouden et al., 2015), further suggesting an involvement of cilia defects in HPE.

More generally, our study of the ciliopathy gene mutant *Rpgrip1l/Ftm* calls for further examination of ciliary and ciliopathy genes in human neurodevelopmental diseases associated with SHH signaling defects.
